# Fluorescence as a Quantitative Indicator of Cariogenic Bacteria During Chemo-Mechanical Caries Excavation with BRIX 3000 in Primary Teeth

**DOI:** 10.3390/jfb16120453

**Published:** 2025-12-06

**Authors:** Zornitsa Lazarova, Raina Gergova, Nadezhda Mitova

**Affiliations:** 1 Department of Pediatric Dentistry, Faculty of Dental Medicine, Medical University—Sofia, 1 Georgi Sofiyski St., 1431 Sofia, Bulgaria; z.lazarova@fdm.mu-sofia.bg; 2 Department of Medical Microbiology, Medical Faculty, Medical University—Sofia, 2 “Zdrave” Str., 1431 Sofia, Bulgaria; r.gergova@medfac.mu-sofia.bg

**Keywords:** minimally invasive dentistry, chemo-mechanical caries excavation, Brix 3000, fluorescence-aided caries excavation, primary teeth, cariogenic microorganisms, *Streptococcus mutans*

## Abstract

Minimally invasive treatment is increasingly successful in managing carious lesions in primary teeth, owing to the regenerative capacity of the dental pulp and the possibility to influence the pulp–dentin complex. Chemo-mechanical caries excavation (CME) with Brix 3000, a papain-based enzymatic agent, allows selective removal of infected dentin while preserving affected dentin for potential remineralization. Fluorescence-aided caries excavation (FACE) enables visualization of porphyrins produced by cariogenic microorganisms, guiding selective dentin removal. In this study, 42 children aged 4–7 years with ICDAS II code 05–06 lesions in primary molars were treated, and the correlation between fluorescence intensity and cariogenic microbial load was evaluated. CME was performed using Brix 3000, and residual dentin was categorized by fluorescence as red, red with pale-pink areas, pale-pink, or non-fluorescent. Microbiological samples were collected pre- and post-excavation, cultured under standardized laboratory conditions, and quantitatively analyzed. Results showed that higher fluorescence intensity corresponded to increased presence of *S. mutans* (ρ = 0.945, *p* < 0.001), while other species were present in lower quantities. CME with Brix 3000 significantly reduced microbial load, and fluorescence reliably indicated areas requiring removal. These findings demonstrate that combining FACE with Brix 3000 allows precise, minimally invasive caries removal in primary teeth, providing an objective method to guide tissue-preserving excavation while effectively controlling cariogenic microorganisms.

## 1. Introduction

Minimally invasive treatment has been widely and successfully applied for the management of carious lesions in pediatric patients. This approach is underpinned by current understanding of the regenerative and reparative potential of the dental pulp, as well as the ability to modulate the pulp–dentin complex during clinical interventions [1,2].

The concept of minimally invasive therapy emphasizes the selective removal of infected and irreversibly denatured dentin, aiming to preserve structurally compromised yet remineralizable dentin [3]. This is particularly important in primary teeth, where anatomical constraints and thinner dentinal walls increase the risk of inadvertent pulp exposure during conventional cavity preparation [4,5]. In this context, chemo-mechanical caries excavation (CME) has emerged as a tissue-preserving and clinically effective alternative to conventional mechanical removal, particularly when enzymatic agents such as Brix 3000 are employed [6,7].

Brix 3000 is an enzymatic agent based on the proteolytic enzyme papain, which is bio-encapsulated using proprietary E.B.E. technology (Encapsulating Buffer Emulsion) to stabilize the enzyme and enhance its proteolytic activity. This confers improved collagen degradation in carious dentin, higher antibacterial and antifungal efficacy, and a pronounced antiseptic effect on the treated tissues [8,9]. Compared to conventional mechanical excavation, enzymatic methods facilitate the preservation of dentin that can undergo remineralization, reducing the risk of pulp exposure and unnecessary removal of sound tissue.

Careful control during cavity preparation is critical within minimally invasive treatment to avoid “overpreparation,” particularly in primary teeth due to their smaller anatomical dimensions, which increase the risk of unnecessary pulp exposure [4,5]. Fluorescence-aided caries excavation (FACE) offers a non-invasive method to distinguish infected from affected dentin by visualizing porphyrins—microbial metabolic by-products that emit red fluorescence under violet light (405 nm). When the carious lesion floor is illuminated, porphyrins indicate areas of infected dentin requiring removal, whereas healthy dentin does not fluoresce. This approach allows selective elimination of infected dentin while preserving affected dentin, which retains the potential for remineralization [10,11,12].

Although fluorescence-guided excavation is increasingly advocated, there is limited evidence on the direct relationship between fluorescence intensity and microbial load, particularly for key cariogenic species such as Streptococcus mutans and *Lactobacillus* spp. Clarifying this relationship is essential to ensure that FACE not only guides tissue-preserving excavation but also effectively reduces cariogenic microbial presence. Addressing this knowledge gap is particularly relevant for chemo-mechanical excavation with Brix 3000 in primary teeth, as it may optimize clinical decision-making, maximize tissue conservation, and potentially minimize pulp-related complications in pediatric patients. Therefore, the present study aimed to investigate the correlation between fluorescence intensity and the quantity of cariogenic microorganisms in dentin carious lesions of primary teeth treated with Brix 3000, providing critical insights into the efficacy of fluorescence-guided minimally invasive approaches and their potential to enhance precision and safety in pediatric dental care.

## 2. Materials and Methods

### 2.1. Study Population

The investigation is a prospective observational and non-interventional microbiological–clinical correlation study. A total of 42 children aged 4 to 7 years were included in this clinical study. Inclusion criteria were as follows:

- Clinically healthy children with at least one approximal or occlusal carious lesion on a primary first or second molar, corresponding to ICDAS II code 05 or 06.
- Absence of spontaneous pain.
- Absence of nocturnal pain.
- Absence of periapical alterations.
- At least one year remaining until physiological exfoliation of the tooth.

All parents of the participating children provided written informed consent. The study was conducted in accordance with national and international ethical standards for clinical research and was approved by the Ethics Committee (KENIMUS Protocol No. 05/20 February 2019). All materials and data generated in this study are available from the corresponding author upon reasonable request.

### 2.2. Chemo-Mechanical Excavation Procedure

All carious lesions were treated using chemo-mechanical caries excavation with Brix 3000 (Brix S.R.L., Carcarañá, Santa Fe, Argentina). The procedure included the following steps: lesion exposure, removal of carious dentin from the enamel-dentin junction (EDJ) and cavity walls, conservative excavation of dentin from the cavity floor, close to the pulp, in accordance with CME principles. Carious tissue was completely removed from the cavity walls, while the cavity floor was treated to preserve affected dentin that could undergo remineralization.

Brix 3000 was applied according to the manufacturer’s instructions: a pea-sized amount was placed into the carious lesion, left for 2 min, and carious dentin was removed using a manual crescent-shaped excavator—three different sizes were used (Excavator Diam 1.4 L, 2 L, 2.5 L; Koine Italia snc, Milan, Italy), depending on lesion size. Each stage of excavation was monitored using fluorescence with the ProFace W&H system (Bürmoos, Austria, W&H Dentalwerk Bürmoos GmbH) at a wavelength of 405 nm. During observation, protective glasses with a filter passing wavelengths up to 500 nm were used.

Fluorescence on the cavity floor was assessed relative to cavity walls that had been cleaned to sound dentin and exhibited no fluorescence, ensuring a baseline for comparison. The number of observations per lesion was recorded, and fluorescence intensity was graded visually.

### 2.3. Dentin Evaluation

The degree of destruction of different dentin types was assessed using Bjorndal’s visual-tactile criteria [13], as well as the following fluorescence criteria:

- *Infected dentin*: intense red or dark red fluorescence encompassing the entire lesion.
- *Partially infected dentin*: pink fluorescence with localized red areas in the near-pulp dentin.
- *Affected dentin*: pale pink fluorescence in isolated areas of the cavity floor, with the remaining areas showing no fluorescence.
- *Sound dentin*: absence of fluorescence.

In accordance with the ICCC (International Caries Consensus Collaboration), tactile sensation was considered the most important criterion for assessing dentin hardness. Dentin was categorized as soft (deforms under pressure, removed with minimal effort), leathery (resists slight pressure, removed easily but with some force), firm (resistant, requiring force to remove), or hard (requires considerable force; probe passage produces a distinct scratching sound) [14].

The rationale for selecting Bjorndal criteria was to standardize dentin classification and ensure reproducibility across examiners. Each lesion was independently evaluated by two calibrated operators. The fluorescence categories were assessed by the operators through certified filter goggles, which reliably display the full red–pink fluorescence spectrum. At the time of the study, no suitable optical filter for the camera system was available, so subtle gradations (pale pink vs. red) could not be accurately captured in photographs. Consequently, classification relied exclusively on direct clinical visualization rather than photographic documentation.

### 2.4. Microbiological Sampling and Analysis

A total of 84 dentin samples with different degrees of destruction were collected before and after chemo-mechanical excavation with Brix 3000, as follows:

- 42 samples from infected dentin (pre-excavation).
- 22 samples from partially infected dentin (post-excavation).
- 10 samples from affected dentin (post-excavation).
- 10 samples from sound dentin (post-excavation).

Dentin sampling was performed only after the complete removal of Brix 3000. Before sample collection, the cavity floor was thoroughly irrigated with sterile saline to eliminate all gel remnants and gently air-dried. Because Brix 3000 is fully water-soluble and retains enzymatic activity only within denatured collagen, irrigation completely inactivates and removes the material, ensuring that no papain or vehicle components remained on the dentin surface at the time of sampling. Samples were collected aseptically using a sterile excavator. Collected material was placed in sterile Eppendorf tubes with transport medium provided by the laboratory, maintained at room temperature, and delivered to the microbiology laboratory within 3–4 h.

Samples were plated on blood agar, selective Lactobacillus agar, and Brain-Heart Infusion broth, incubated at 36 °C with CO_2_ for 24–48 h. If insufficient growth occurred on solid media, enrichment broth was replated onto fresh agar. Pure cultures were isolated from morphologically suspicious colonies. Isolates were identified and quantified in CFU/mL. All laboratory procedures followed standard aseptic techniques, including the use of gloves, masks, and sterilized instruments, to minimize contamination.

**Figure 1:**
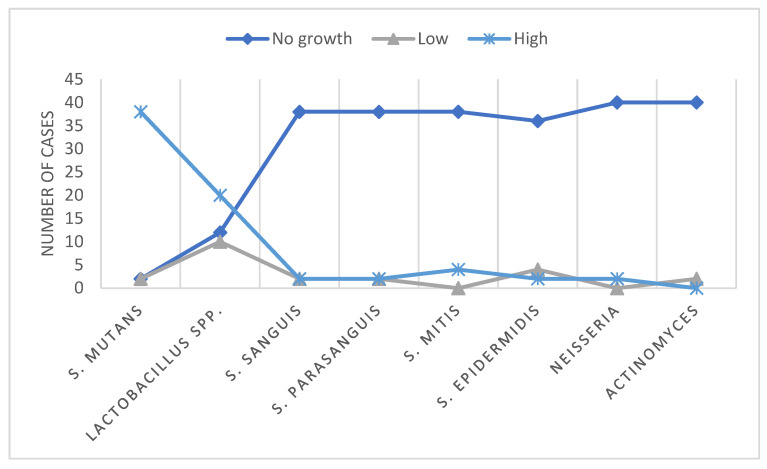
Distribution of colony-forming units (CFU/mL) for cariogenic microorganisms in red-fluorescing dentin before chemo-mechanical excavation. Samples were classified into three CFU categories: no growth (0 CFU/mL), low (10^3^–10^4^ CFU/mL), and high (10^5^–10^7^ CFU/mL). Bars represent the number of samples per category for each microorganism.

Quantitative analysis of bacterial load was performed by counting colony-forming units (CFU) per mL for all isolated cariogenic species (*S. mutans*, *Lactobacillus* spp., *S. sanguis*, *S. parasanguis*, *S. mitis*, *S. epidermidis*, *Neisseria* spp., *Actinomyces* spp.). For visualization in Figure 1, CFU counts were categorized as follows: no growth (0 CFU/mL), low (10^3^–10^4^ CFU/mL), and high (10^5^–10^7^ CFU/mL). These log-based thresholds were chosen based on literature values and allow comparison of microbial load across different species.

### 2.5. Statistical Analysis

Data were analyzed using IBM SPSS version 19.0 and MS Excel 2019. The following methods were applied to objectively evaluate results:

- Descriptive analysis—frequency distribution of studied variables presented in tables.
- Pearson Chi-Square test (χ^2^)—to assess associations between categorical variables.
- Spearman’s rank correlation coefficient (Spearman’s rho)—used to evaluate relationships between variables with non-normal distribution or ordinal data. Correlation coefficients of 0.1–0.3 were considered weak, 0.3–0.5 moderate, and >0.5 strong.
- Graphical representation of statistical results was generated to facilitate interpretation.

The choice of Spearman correlation was based on the ordinal nature of fluorescence intensity and microbiological load, allowing non-parametric assessment of relationships between these variables.

## 3. Results

### 3.1. Microbial Distribution Before Chemo-Mechanical Excavation

Figure 1 shows the distribution of CFU counts for all cariogenic microorganisms in dentin exhibiting red fluorescence prior to chemo-mechanical excavation. *S. mutans* predominated in most samples, frequently falling into the high CFU category (10^5^–10^7^ CFU/mL). *Lactobacillus* spp. were detected at lower frequencies, mostly in the low CFU range (10^3^–10^4^ CFU/mL). Other species, including *S. sanguis*, *S. parasanguis*, *S. mitis*, *S. epidermidis*, *Neisseria* spp., and *Actinomyces* spp., were observed only sporadically, generally in the low CFU category. These results indicate that intense red fluorescence corresponds to a higher diversity and quantity of cariogenic microorganisms.

- *S. mutans*—predominates in quantity, highlighting its key role in caries development (90.48%).
- *Lactobacillus* spp.—present in lower amounts, contributing to lesion progression (47.62%).
- Other species:
  - *S. sanguis* (4.76%)
  - *S. parasanguis* (4.76%)
  - *S. mitis* (9.52%)
  - *S. epidermidis* (4.76%)
  - *Neisseria* spp. (4.76%)
  - *Actinomyces* spp. (0%)

These were isolated only in some cases of red fluorescence, indicating a secondary role in dentin demineralization.

### 3.2. Microbial Distribution After Chemo-Mechanical Excavation

**Figure 2:**
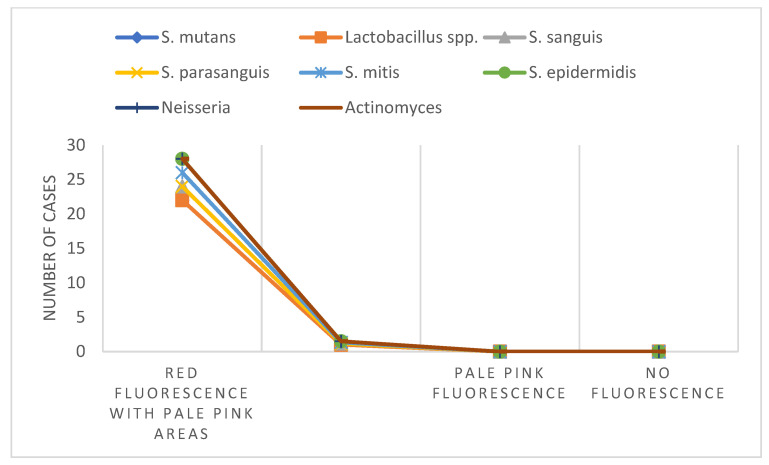
Distribution of isolated microbial species according to fluorescence intensity after chemo-mechanical excavation with Brix 3000. Numbers indicate the samples (n) in which each microorganism was detected within the respective fluorescence category: red (infected dentin), red with pale-pink areas (partially infected dentin), pale pink (affected dentin), and no fluorescence (sound dentin).

Figure 2 shows the distribution of isolated microorganisms according to fluorescence intensity after chemo-mechanical excavation with Brix 3000. The number of samples (n) in which each microorganism was detected is indicated for each fluorescence category.

After chemo-mechanical excavation with Brix 3000, both microbial count and diversity decreased. *S. mutans* was consistently isolated in all samples with red fluorescence containing pale-pink areas, demonstrating its resilience and confirming the reliability of fluorescence-guided excavation for identifying residual microbial presence in dentin with lower degrees of destruction. Other species (*S. sanguis*, *S. mitis*, *S. epidermidis*) were detected only sporadically, exclusively in red fluorescence with pale-pink areas. No microorganisms were recovered from dentin exhibiting pale-pink or absent fluorescence.

Given that *Streptococcus mutans* is the primary microorganism isolated in the highest quantities and remains relatively stable over time, we focus our analysis on this species.

### 3.3. Relative Proportion of S. mutans

To further clarify the relationship between *S. mutans* presence and dentin fluorescence intensity, Table 1 presents the number of samples (n) and corresponding percentages in which *S. mutans* was detected across the different fluorescence categories during chemo-mechanical excavation with Brix 3000. This presentation allows clear interpretation of both the prevalence and relative quantity of *S. mutans* in relation to the fluorescence-guided excavation stages.

*S. mutans* was isolated in high amounts (1 × 10^5^–10^7^ CFU/mL) in 45.2% of cases, in lower amounts (1 × 10^3^–10^4^ CFU/mL) in 28.6%, and no growth was detected in 26.2% of samples. The presence and quantity of *S. mutans* decreased with decreasing fluorescence intensity, confirming the reliability of fluorescence-guided excavation for tissue preservation. Chi-square analysis confirmed that higher fluorescence levels were significantly associated with higher *S. mutans* counts (χ^2^(6) = 146.061, *p* < 0.001).

### 3.4. Correlation Between S. mutans Quantity and Fluorescence Intensity

Table 2 presents the Spearman correlation between *S. mutans* quantity and fluorescence intensity.

Observation: Spearman correlation analysis showed a strong positive correlation between *S. mutans* quantity and fluorescence intensity (ρ = 0.945, *p* < 0.001). This indicates that higher fluorescence intensity corresponds to higher amounts of *S. mutans*, confirming the efficacy of fluorescence-guided excavation in detecting and reducing cariogenic microbial load.

## 4. Discussion

The present study demonstrates that the combined use of Brix 3000 chemo-mechanical caries excavation and fluorescence-guided excavation control provides a biologically rational and clinically effective approach for managing dentin carious lesions in primary teeth. The data clearly show that fluorescence intensity closely corresponds to the degree of microbial contamination within dentin, both at baseline and during progressive removal of infected tissue. This relationship highlights the potential of fluorescence to serve not only as a visual indicator of caries activity, but also as an objective biomarker reflecting the underlying microbial load, which is of particular importance in minimally invasive pediatric dentistry [1,2,3].

Spearman correlation analysis clearly demonstrates that higher fluorescence intensity corresponds to a greater quantity of *S. mutans*, the primary cariogenic microorganism (*p* < 0.01). These results indicate that fluorescence-guided excavation can be successfully applied for the treatment of dentin carious lesions in primary teeth using Brix 3000 [10]. Furthermore, the findings highlight the resilience of *S. mutans*, which was isolated in 90.48% of cases in the highest quantities among all microorganisms, corresponding to red fluorescence, a marker of infected dentin. Importantly, these microbial levels were not associated with pulp exposure or post-operative sensitivity in any of the treated teeth. The selective enzymatic action of Brix 3000, combined with careful fluorescence-guided excavation, allowed effective reduction in bacterial load while preserving pulp vitality, minimizing the risk of post-operative discomfort. In addition to *S. mutans*, fluorescence also allowed the detection of other microorganisms, such as *Lactobacillus* spp., the second most abundant microorganism, present in high amounts (1 × 10^5^–10^7^ CFU/mL) in nearly 48% of cases. Red fluorescence also revealed the occasional presence of *S. sanguis*, *S. parasanguis*, *S. mitis*, *S. epidermidis*, *Neisseria* spp., and *Actinomyces* spp., albeit in lower quantities. Following chemo-mechanical excavation with Brix 3000 to different types of residual dentin, this microbial diversity noticeably decreased. In areas showing red fluorescence with pale-pink regions, indicative of partially infected dentin, *S. mutans* was consistently isolated, even in lower quantities (1 × 10^3^–10^4^ CFU/mL). In the same range, *S. sanguis*, *S. mitis*, and *S. epidermidis* were occasionally detected. These findings further support that fluorescence can reliably detect the presence of cariogenic microorganisms even at lower concentrations, guiding selective and tissue-preserving excavation [15,16,17,18].

Despite these promising results, there are currently no precise criteria to determine the exact endpoint of caries excavation. The selective removal of infected dentin using Brix 3000 is based on its enzymatic mechanism, which targets denatured collagen in carious dentin while sparing healthy tissue. In infected dentin, the absence of the anti-protease α-1-antitrypsin allows papain to degrade collagen, whereas in sound dentin, the presence of this anti-protease deactivates papain, preventing unnecessary tissue removal. This biochemical inhibition provides absolute assurance that BRIX3000^®^ cannot dissolve sound or remineralizable dentin, as papain becomes inactive in the presence of intact organic matrix and α-1-antitrypsin. Unlike dye-based detectors, which stain less-mineralized collagen regardless of bacterial invasion, the enzymatic activity of BRIX3000^®^ is strictly limited to irreversibly denatured collagen found only in infected dentin.

Fluorescence-guided assessment serves as a visual adjunct, with intense red indicating areas of infected dentin that require removal, red with pale-pink highlighting partially infected regions, and pale-pink or non-fluorescent areas corresponding to dentin that can be preserved for potential remineralization. However, fluorescence does not directly correspond to enzymatic degradability, as it reflects increased porosity, organic matrix content, and bacterial metabolites rather than the presence or absence of α-1-antitrypsin. Therefore, areas showing persistent fluorescence may not always be susceptible to papain activity. However, certain limitations must be acknowledged: the intensity of fluorescence can be influenced by moisture or pigmentation, the penetration depth of the fluorescent signal is limited, and interpretation remains operator-dependent, which may in some cases lead to removal of remineralizable dentin. This may lead to false-positive fluorescence signals and, if relied upon without considering enzymatic selectivity and tactile hardness, may result in over-preparation of dentin that still possesses remineralization potential. This integrated approach, combining enzymatic selectivity with fluorescence guidance, provides a reliable method to achieve conservative caries excavation while minimizing the risk of excessive removal of affected or healthy dentin [19,20].

In clinical practice, selective removal of infected dentin can also be guided by caries detector dyes. Historically, Fusayama introduced staining techniques to differentiate infected from affected dentin using fuchsin solutions, showing that dye penetration roughly corresponds to microbial invasion, although deeper bacterial spread may exceed dye penetration. Modern evidence indicates that such dyes primarily stain the organic matrix of less-mineralized dentin rather than microorganisms themselves. Their specificity is limited, as even sound dentin with higher organic content may take up the dye, potentially leading to over-preparation. FACE, in contrast, provides real-time visual feedback on microbial activity and dentin porosity, complementing tactile assessment and offering higher selectivity for infected tissue. Therefore, fluorescence-guided excavation may serve to replace, complement, or be used in combination with traditional dye-based methods, providing more precise guidance for conservative caries removal [19,21,22,23].

The combined use of Brix 3000 and fluorescence-guided assessment in this study ensured conservative tissue removal without unnecessary loss of remineralizable dentin. In this protocol, fluorescence served as an adjunctive visual indicator, helping to differentiate infected from partially infected and potentially preservable dentin. While fluorescence highlights areas of increased porosity and bacterial metabolites, it remains an adjunctive tool rather than a standalone determinant of excavation endpoint. For this reason, fluorescence should complement, rather than replace, tactile hardness assessment. Integrating both methods provides a balanced and reliable strategy for selective excavation in primary teeth, minimizing the risk of excessive removal of affected but remineralizable dentin [24,25,26,27].

Visual-tactile criteria are considered the gold standard for assessing dentin after excavation, with the hardness of the residual dentin regarded as the primary indicator. However, this assessment is inherently subjective, highlighting the need for additional objective methods to complement visual-tactile evaluation [27,28].

The literature contains numerous studies demonstrating the need for fluorescence-guided control during caries excavation, as well as the role of microorganisms in the progression of the carious process. Lennon et al. examined 66 primary molars, assessing residual dentin using fluorescence-guided control, dye staining, and visual-tactile evaluation. The authors concluded that fluorescence-guided control leads to more conservative removal of carious dentin, which is particularly important in primary teeth, where the risk of unintended pulp exposure is high due to superficially exposed pulp horns [10]. Other studies have reached similar conclusions, showing that fluorescence can be successfully applied during excavation and helps limit unnecessary removal of hard dental tissues [11,29]. All these findings support our results and demonstrate that fluorescence is an effective method for distinguishing different types of residual dentin, clearly indicating the point at which excavation should be stopped.

Numerous studies have also demonstrated the antimicrobial activity of papain, the main component of Brix 3000. Goyal et al. compared the conventional mechanical preparation method with the enzymatic approach in 25 children aged 5 to 9 years. Microbiological samples were collected before and after caries excavation, and the results showed a significant reduction in both the number and quantity of *S. mutans* and *Lactobacillus* spp. [30]. Similar findings have been reported by other authors, who also observed reductions in *S. mutans* and *Lactobacillus* spp. [11,31]. These results support our findings. The literature also indicates that papain can inhibit bacterial growth by cleaving peptide bonds in microorganisms into dipeptides and amino acids. Papain, a member of the sulfhydryl protease family, possesses a sulfhydryl residue at its active site that interacts with the bacterial cell wall and cytoplasmic membrane, which underlies its antimicrobial effect [31]. This evidence highlights the dual role of Brix 3000 in minimally invasive dentistry: enzymatic tissue removal and antimicrobial activity, enhancing both clinical precision and microbial control.

Although numerous studies in the literature have demonstrated the benefits of fluorescence-guided excavation and the effectiveness of Brix 3000, as well as the role of microorganisms—particularly *S. mutans*—in caries progression, there is a lack of studies directly linking all three factors. Our study provides new evidence connecting fluorescence intensity, microbial load, and the efficacy of Brix 3000, supporting its use as a comprehensive minimally invasive approach in primary teeth.

Taken together, these results not only demonstrate the reliability of fluorescence as a biomarker for microbial load but also provide practical insights for enhancing clinical decision-making in pediatric dentistry. These findings support the integration of fluorescence-guided CME with Brix 3000 into routine pediatric dental practice, as this approach allows selective removal of infected dentin, preserves remineralizable tissue, and reduces the risk of pulp exposure. Incorporating fluorescence monitoring provides an objective guide that complements visual-tactile assessment, enhancing precision and patient safety.

Despite the promising results, several limitations of this study should be acknowledged. The relatively small sample size may limit the generalizability of the findings, and variability in lesion depth and patient age could influence both fluorescence readings and microbial detection. The visual-tactile assessment of residual dentin, although calibrated, still retains an inherent subjective component. Furthermore, the study focused on culturable microorganisms, which may underestimate the full diversity of the cariogenic microbiome, suggesting that future research should incorporate molecular methods for a more comprehensive analysis. Regarding sampling, although Brix 3000 is fully water-soluble and the cavities were thoroughly rinsed with sterile saline and gently dried before collection, a theoretical risk of minimal dilutional effects on dentin chips cannot be completely excluded. However, the short contact time and the requirement for denatured collagen to sustain papain activity make any influence on bacterial viability or CFU counts highly unlikely.

Another limitation concerns fluorescence documentation. At the time of the study, appropriate optical filters for photographic recording of FACE images were not available, and fluorescence categories were evaluated visually through protective filter glasses. As a result, the subtle transitions between pale-pink, red, and intense red fluorescence could not be reliably captured for inclusion as clinical figures. Future studies employing standardized fluorescence imaging systems would strengthen the visual documentation and improve reproducibility.

Regarding future research, larger and more diverse populations should be evaluated, molecular methods could be incorporated to assess the full spectrum of cariogenic microorganisms, and long-term clinical outcomes—including lesion recurrence, pulp health, and remineralization potential of preserved dentin—should be investigated. These efforts will further validate the integration of fluorescence-guided CME into routine pediatric dental practice and contribute to refining guidelines for minimally invasive caries management.

## 5. Conclusions

Fluorescence-guided control during chemo-mechanical excavation with Brix 3000 in primary teeth allows precise, selective, and conservative removal of carious dentin, effectively reducing microbial load while preserving pulp vitality, supporting tissue preservation, and enhancing patient safety.

## Figures and Tables

**Table 1:** Relative proportion of *S. mutans* in dentin with varying fluorescence intensity during excavation with Brix 3000.

Quantity *S. mutans*	No Fluorescence		Pale-Pink Fluorescence		Red with Pale-Pin		Red Fluorescence		Total	
	n	%	n	%	n	%	n	%	n	%
No growth	10	11.9%	10	11.9%	0	0%	2	2.4%	22	26.2%
1.10^3–4^	0	0%	0	0%	22	26.2%	2	2.4%	24	28.6%
1.10^5–7^	0	0%	0	0%	0	0%	38	45.2%	38	45.2%
Total	10	11.9%	10	11.9%	22	26.2%	42	50%	84	100%
χ^2^ (6) = 146.061 ^a^ *p* < 0.001										

Note: Percentages are calculated relative to the total number of samples (n = 84). ^a^—statistically significant difference.

**Table 2:** Spearman correlation between *S. mutans* quantity and fluorescence type.

	*S. mutans* Quantity	Type of Fluorescence
*S. mutans* quantity	1.000	ρ = 0.945 **
Type of fluorescence		1.000

ρ—Spearman’s correlation coefficient. *p* < 0.001 (2-tailed). N = 84. ** Correlation is significant at the *p* < 0.01 level.

## Data Availability

The original contributions presented in the study are included in the article, further inquiries can be directed to the corresponding author.

## Abbreviations

The following abbreviations are used in this manuscript:

CME	Chemo-mechanical caries excavation
FACE	Fluorescence-aided caries excavation
ICDAS II	International Caries Detection and Assessment System II
